# Statistically efficient association analysis of quantitative traits with haplotypes and untyped SNPs in family studies

**DOI:** 10.1186/s12863-020-00902-x

**Published:** 2020-09-07

**Authors:** Guoqing Diao, Dan-yu Lin

**Affiliations:** 1 Department of Biostatistics and Bioinformatics, The George Washington University, Washington, District of Columbia USA; 2 Department of Biostatistics, University of North Carolina at Chapel Hill, Chapel Hill, North Carolina USA

**Keywords:** Complex diseases, EM algorithm, Gene-environment interactions, Haplotype analysis, Hardy-Weinberg equilibrium, Unphased genotype, Variance-component models

## Abstract

**Background:**

Associations between haplotypes and quantitative traits provide valuable information about the genetic basis of complex human diseases. Haplotypes also provide an effective way to deal with untyped SNPs. Two major challenges arise in haplotype-based association analysis of family data. First, haplotypes may not be inferred with certainty from genotype data. Second, the trait values within a family tend to be correlated because of common genetic and environmental factors.

**Results:**

To address these challenges, we present an efficient likelihood-based approach to analyzing associations of quantitative traits with haplotypes or untyped SNPs. This approach properly accounts for within-family trait correlations and can handle general pedigrees with arbitrary patterns of missing genotypes. We characterize the genetic effects on the quantitative trait by a linear regression model with random effects and develop efficient likelihood-based inference procedures. Extensive simulation studies are conducted to examine the performance of the proposed methods. An application to family data from the Childhood Asthma Management Program Ancillary Genetic Study is provided. A computer program is freely available.

**Conclusions:**

Results from extensive simulation studies show that the proposed methods for testing the haplotype effects on quantitative traits have correct type I error rates and are more powerful than some existing methods.

## Background

With the advances in high-throughput genotyping technologies and the availability of dense SNP maps across the human genome [1], haplotype-based association analysis plays an increasingly important role in mapping genes that influence complex human diseases. Haplotypes, which are specific combinations of alleles at several tightly linked SNPs on a chromosome, incorporate the linkage disequilibrium information and pertain to the functional properties of proteins through the amino acids sequences. Association analysis based on haplotypes tends to be more powerful than the analysis of individual SNPs, especially when the causal SNPs are not directly typed or when multiple mutations occur in the *cis* position [2–6].

Standard genotyping procedures only measure unphased genotypes rather than haplotypes. Haplotypes are ambiguous if the genotypes of a subject are heterozygous at more than one marker locus. The ambiguity of the gametic phase information poses a major challenge in the haplotype analysis. For population-based studies with unrelated individuals, a number of methods have been developed to estimate haplotype frequencies or infer individual haplotypes from unphased genotype data [7–12] and to make inference about the effects of haplotypes on disease phenotypes [13–22].

Family studies are more attractive than population-based studies because family data reduce the ambiguity of haplotypes and are less prone to spurious associations caused by population admixture and stratification. Several methods have been developed to estimate haplotype frequencies or infer individual haplotypes from unphased genotype data for general pedigrees, including HAPLORE [23], GENEHUNTER [24], PedPhase [25], and MERLIN [26]. Zhang and Zhao [27] demonstrated through simulation studies that HAPLORE and MERLIN had comparable performance and outperformed the other two methods.

Several methods have been developed for the haplotype association analysis in family studies. Horvath et al. [28] extended the method of Rabinowitz and Laird [29] to multiple markers and proposed a haplotype version of family-based association tests (FBAT). The haplotype FBAT estimates the haplotype frequencies by the expectation-maximization (EM) algorithm [30] under Hardy-Weinberg equilibrium. A score statistic is then constructed in the same manner as the original FBAT except that the genotype score is coded as a weighted sum of haplotype scores, the weight being the conditional probability of a particular haplotype configuration given that it is compatible with the unphased genotype. The haplotype FBAT is computationally simple and can provide either haplotype-specific tests or multi-haplotype tests. However, this method is limited to nuclear families without covariates, does not account for within-family trait correlations, and does not estimate genetic effects. Furthermore, it discards the parental phenotype information and thus may cause substantial loss of power. Dudbridge [31] proposed a retrospective likelihood approach for the association analysis for nuclear families and unrelated subjects with missing genotype data. The retrospective likelihood is based on the probability of observing the parental and offspring genotypes, given the trait values of the all the children in a nuclear family. Other related work includes the family-based association test for dichotomous traits [32], the extension of haplotype FBAT to multiple phenotypes [33], and a Bayesian regression method [34].

Missing genotype data are inevitable in genetic association studies. For example, some study subjects may have missing genotypes at certain SNP loci due to assay failures. Another form of missing data arises when the investigators are interested in untyped SNPs, i.e., the SNPs that are not on the genotyping platform used in the study and thus missing on all study subjects. Haplotypes provide an effective way of inferring the missing genotypes at a particular SNP from the observed genotypes of neighboring SNPs. Lin et al. [35] developed efficient likelihood-based methods to deal with missing genotype data in case-control studies. For family studies, Burdick et al. [36] and Chen and Abecasis [37] imputed the missing genotype values by their expected values via the Elston-Steward or Lander-Green algorithm. Both methods require that at least some members of a family have non-missing genotype data so that they can be used to estimate the conditional distribution of the missing genotypes for other members of the family. These methods cannot be used when the genotype data of the entire family are missing and thus cannot handle untyped SNPs.

In this paper, we present an efficient likelihood-based approach to studying the associations between haplotypes and quantitative traits. This approach estimates the haplotype frequencies and the haplotype effects on the quantitative trait simultaneously. It is very efficient in dealing with missing genotype data. In addition, it allows departures from Hardy-Weinberg equilibrium, accounts for within-family trait correlations, and accommodates general pedigrees with arbitrary patterns of missing data. We characterize the effects of haplotypes on the quantitative trait by a linear regression model with random effects and derive the corresponding likelihood function. We develop efficient likelihood-based estimation and testing procedures. Extensive simulation studies show that the new methods perform well in realistic scenarios. An application to family data from the Childhood Asthma Management Program (CAMP) Ancillary Genetic Study [38] is provided.

## Results

### Simulation studies

We conducted extensive simulation studies to assess the performance of the new methods in realistic settings. We simulated SNP genotypes according to the haplotype distribution observed in the CEU sample of the HapMap. The inbreeding coefficient *ρ* was set to 0.02. We generated the quantitative trait values from model (2) in the Methods section with a potentially causal haplotype or SNP. For each scenario, we generated 10,000 data sets, each of which contains 100 nuclear families with two parents and two children.

In the first set of simulation studies, we evaluated the performance of the new method for haplotype association analysis. We were particularly interested in SNPs 20-24 on chromosome 18 of the CEU sample in the HapMap genomewide data. This set of SNPs was previously considered by Lin et al. [35]. The LD among the 5 SNPs is not particularly strong. The five most common haplotypes are 00000, 00011, 00100, 01000, and 01011, with frequencies 0.1431, 0.1312, 0.1941, 0.1756, and 0.1579, respectively. We generated trait values from the additive model

$$Y_{ij} = \alpha + \beta_{1}\{I(H_{ij1}=h^{*})+I(H_{ij2}=h^{*})\} + \beta_{2}X_{ij} $$

1 $$ +\beta_{3} \{I(H_{ij1}=h^{*})+I(H_{ij2}=h^{*})\} X_{ij}+ g_{ij} + e_{ij}, \\  $$

where the target haplotype *h*^∗^ is 00100 and the environmental variable *X*_*ij*_ is a Bernoulli random variable with 0.3 success probability. The parameters *β*_1_,*β*_2_, and *β*_3_ correspond to the effect of the target haplotype, the effect of the environmental variable, and the haplotype-environment interaction, respectively. We set $\alpha, \sigma _{g}^{2}$, and $\sigma _{e}^{2}$ to 1, 0.5, and 1.0, respectively. For making inference on *β*_1_, we set *β*_3_=0.4 and varied *β*_1_ from 0 to 0.4; for making inference on *β*_3_, we set *β*_1_=0.4 and varied *β*_3_ from 0 to 0.4. For each setting, we considered both the situation of no missing genotypes and the situation with 10% randomly missing genotypes.

Tables 1 and 2 summarize the results for estimating the haplotype effect and haplotype-environment interaction, respectively, while Table 3 presents the results for estimating the haplotype frequencies under *β*_1_=*β*_3_=0.4. The estimators of the haplotype effect and haplotype-environment interaction are virtually unbiased, and so are the estimators of the haplotype frequencies. The variance estimators accurately reflect the true variations of the parameter estimators, and the confidence intervals have correct coverage probabilities. The results of the haplotype frequencies estimates are similar to those obtained from HAPLORE. However, our main objective is to conduct haplotype association analysis and the proposed full information maximum likelihood approach typically yields statistically efficient parameter estimators by the parametric likelihood theory.

**Table 1:** Summary statistics for the estimation of the haplotype effect

Effect size	Missing rate	Bias	SE	SEE	CP
0	0.0	0.001	0.137	0.136	0.949
	0.1	0.000	0.138	0.139	0.949
0.1	0.0	0.001	0.136	0.136	0.950
	0.1	-0.003	0.140	0.139	0.949
0.2	0.0	-0.002	0.136	0.136	0.950
	0.1	-0.002	0.139	0.139	0.949
0.3	0.0	0.002	0.133	0.136	0.955
	0.1	-0.002	0.139	0.139	0.946
0.4	0.0	-0.002	0.136	0.136	0.950
	0.1	-0.002	0.139	0.139	0.945

SE is the sampling standard error of the parameter estimator, SEE is the mean of the standard error estimator, and CP is the coverage probability of the 95% confidence interval

**Table 2:** Summary statistics for the estimation of the haplotype-environment interaction

Effect	Missing				
size	rate	Bias	SE	SEE	CP
0	0.0	0.003	0.237	0.234	0.946
	0.1	0.002	0.241	0.238	0.942
0.1	0.0	0.003	0.237	0.234	0.946
	0.1	0.003	0.241	0.238	0.942
0.2	0.0	0.003	0.237	0.234	0.947
	0.1	0.006	0.241	0.238	0.946
0.3	0.0	0.003	0.237	0.234	0.947
	0.1	0.003	0.241	0.238	0.946
0.4	0.0	0.003	0.237	0.234	0.947
	0.1	0.003	0.241	0.238	0.946

SE is the sampling standard error of the parameter estimator, SEE is the mean of the standard error estimator, and CP is the coverage probability of the 95% confidence interval

**Table 3:** Summary statistics for the estimation of haplotype frequencies

Haplotype	Bias	SE	SEE	CP
00000	-0.0004	0.0181	0.0178	0.943
00011	0.0003	0.0173	0.0172	0.945
00100	0.0000	0.0202	0.0200	0.947
01000	0.0001	0.0197	0.0193	0.943
01011	-0.0003	0.0186	0.0185	0.945

SE is the sampling standard error of the parameter estimator, SEE is the mean of the standard error estimator, and CP is the coverage probability of the 95% confidence interval

**Fig. 1:**
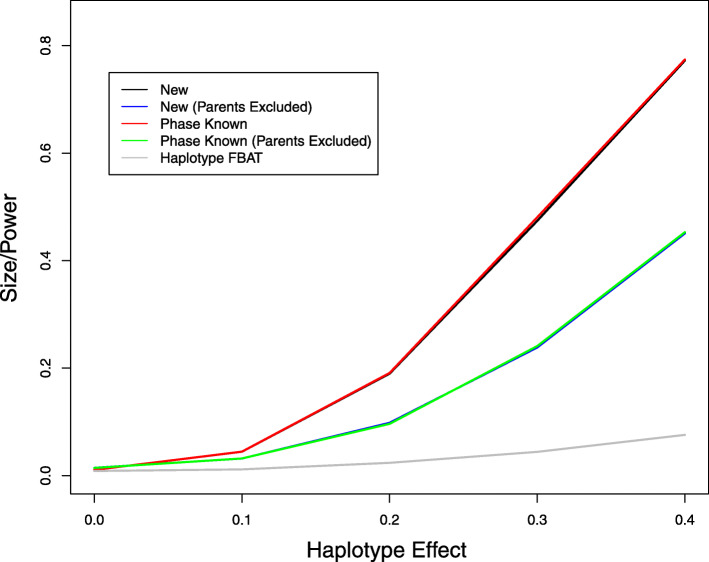
Type I error and power of association tests on haplotype 00100 at the 1% nominal significance level when there are no missing genotype data

**Fig. 2:**
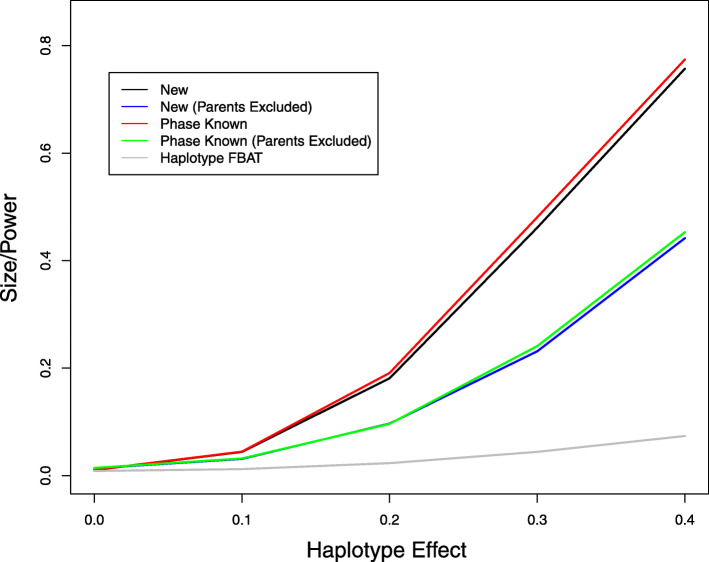
Type I error and power of association tests on haplotype 00100 at the 1% nominal significance level when there are 10% missing genotype data

We also compared the new method to the Haplotype FBAT. Since the latter cannot handle covariates, we set *β*_2_=*β*_3_=0 in model (1). Figures 1 and 2 display the type I error and power of the association tests for the haplotype effect at the nominal significance level of 0.01 without missing data and with 10% missing data, respectively. The new method has the correct type I error and is more powerful than the Haplotype FBAT. The power differences are particularly strong when parental phenotype data are available. The power gain of the new method over the Haplotype FBAT is expected to be even more substantial in the presence of covariate effects. Without missing data, the new method has almost the same power as the ideal case of known haplotypes. The loss of power for the new method caused by missing genotypes is rather moderate, even when there is substantial missingness. These results suggest that the new method can effectively infer the haplotype configuration and is efficient in dealing with missing genotype data.

**Fig. 3:**
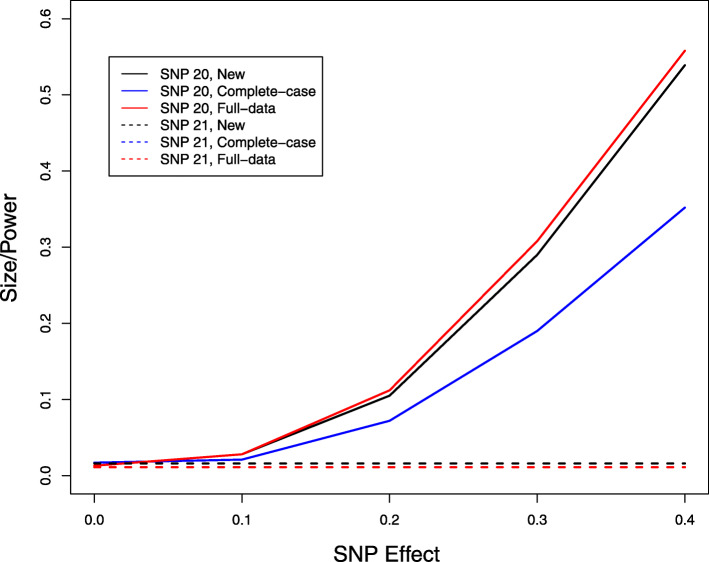
Type I error and power of association tests at SNP 20, which has a causal additive effect on the phenotype, and SNP 21, which is null, at the 1% nominal significance level when there are 10% missing genotype data. For complete-data analysis, all subjects with missing data are removed. For full-data analysis, the missing genotypes are replaced by their true values

**Fig. 4:**
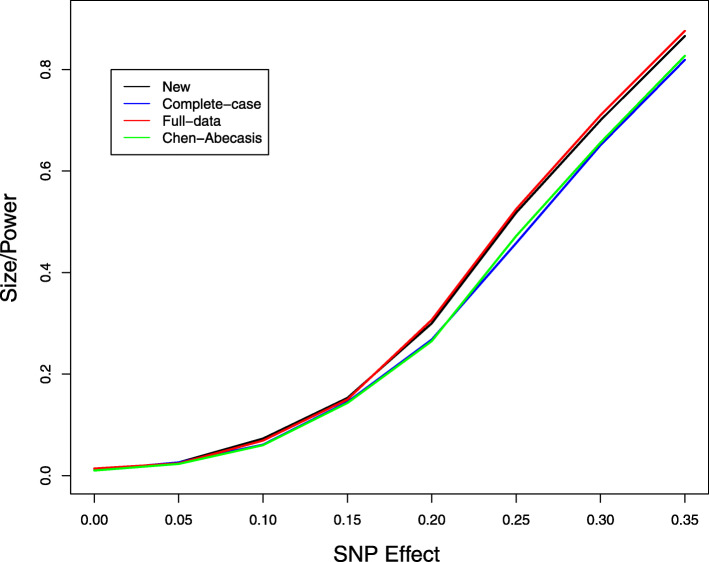
Type I error and power of single-SNP association tests at SNP 20, which has a causal additive effect on the phenotype, at the 1% nominal significance level when there are 10% missing genotype data. For complete-data analysis, all subjects with missing data are removed. For full-data analysis, the missing genotypes are replaced by their true values

**Fig. 5:**
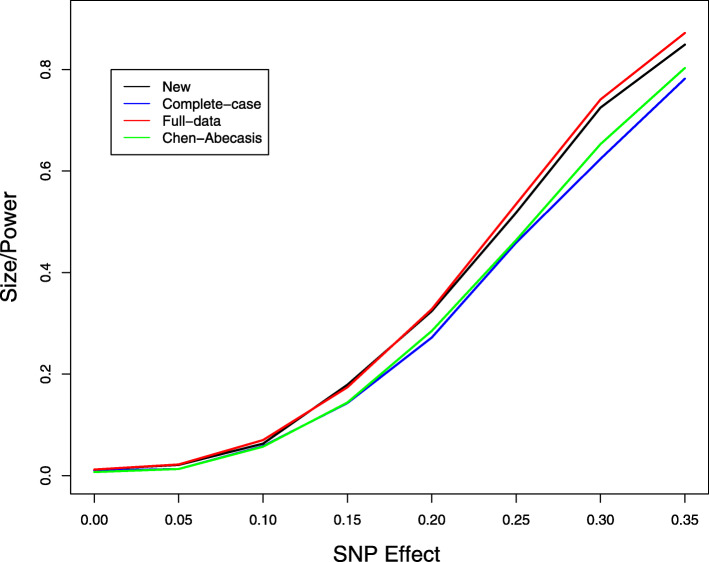
Type I error and power of single-SNP association tests at SNP 20, which has a causal additive effect on the phenotype, at the 1% nominal significance level when there are 20% missing genotype data. For complete-data analysis, all subjects with missing data are removed. For full-data analysis, the missing genotypes are replaced by their true values

We next studied the problem of missing genotype data. We considered the same model as before but set SNP 20 to be causal with an additive effect. We let the genotypes of the 5 SNPs be missing independently with a 10% missing rate and performed multi-SNP analysis by including all 5 SNPs in the model. Figure 3 displays the type I error of the association tests at SNP 21, which is null, and the power of the association tests at SNP 20, which is causal. The new method provides accurate control of the type I error. The improvement of the new method over the complete-case analysis is substantial. Compared to the full-data analysis, the new method has little loss of power. We also performed single-SNP analysis by including only the causal SNP in the model and compared the new method to the imputation method by Chen and Abecasis [37]. Figures 4 and 5 show the size/power curves of the association tests at SNP 20 with missing genotype rates of 10% and 20%, respectively. The new method is substantially more powerful than the imputation approach, especially when the missing rate is high.

**Fig. 6:**
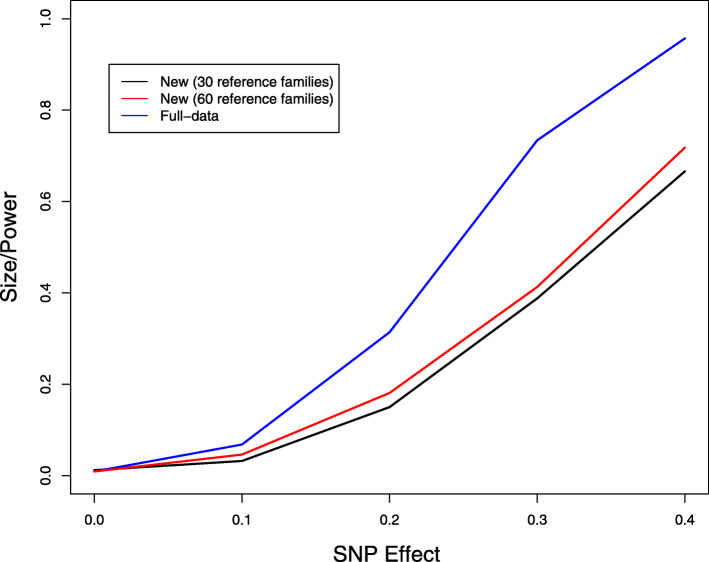
Type I error and power of association tests of untyped SNP 20 at the 1% nominal significance level. For full-data analysis, the missing genotypes are replaced by their true values

We finally studied the problem of untyped SNPs. We considered the same model as in the above simulation studies with missing genotype data. We set the causal SNP 20 to be untyped and performed single-SNP analysis on SNP 20. In addition to the study sample, we generated a reference panel with 30 or 60 nuclear families. As shown in Fig. 6, the new method has proper type I error and reasonable power compared to the ideal full-data analysis. The reference panel of 30 families is almost as informative as that of 60 families.

### CAMP study

We used the new method to study associations between asthma phenotypes and eight SNPs in the Beta2-Adrenergic Receptor (*β*_2_AR) with data from the CAMP Ancillary Genetic Study and compared the results to those of the haplotype FBAT. The CAMP study was a clinical trial of asthmatic children (mild to moderate asthma) who were randomized to three different treatments. The CAMP data set consists of 2,011 individuals in 652 nuclear families. Four percent of the genotypes at eight *β*_2_AR SNPs were missing. Polymorphisms in *β*_2_AR were found to be associated with several asthma phenotypes in previous studies [39, 40]. In this paper, we considered the standardized mean asthma symptom score. Only 573 individuals had non-missing asthma symptom score data, but genotype data for all individuals were used in the model to infer the haplotype configurations. The same data set was previously analyzed in [28].

As shown in Table 4, the new method and the haplotype FBAT identified the same eight haplotypes with frequencies greater than 0.01. The inbreeding coefficient was estimated at 0.04, with a *p*-value 0.005. The Haplotype FBAT did not detect any significant associations, whereas the new method detected significant associations of haplotypes “12211211” and “11211211” with the mean asthma symptom score, the *p*-values being 0.017 and 0.023, respectively. Note that the Haplotype FBAT does not estimate haplotype effects.

**Table 4:** Haplotype-specific association analysis of the asthma symptom score in the CAMP study

New Method						Haplotype FBAT		
Haplotype	Freq.	Effect	LRT	*p*-value		Freq.	Z-stat	*p*-value
11122211	0.369	-0.052	0.739	0.390		0.357	0.904	0.366
12211211	0.345	0.144	5.743	0.017		0.352	-0.308	0.758
11221112	0.183	-0.034	0.182	0.669		0.180	-0.879	0.379
11211211	0.038	-0.377	5.153	0.023		0.037	-1.525	0.127
11221111	0.032	-0.052	0.056	0.813		0.029	1.432	0.152
11221122	0.011	0.223	0.782	0.376		0.011	0.998	0.318

The results presented in Table 4 pertain to the comparison of a target haplotype with all other haplotypes. We also performed an overall test by comparing six most frequent haplotypes to all other haplotypes within the same model. The resultant LR was 11.66 with 6 degrees of freedom, the corresponding *p*-value being 0.07. The chi-square test statistic from the haplotype FBAT was 6.98, with a *p*-value of 0.32.

## Conclusion

Haplotype-based association analysis of quantitative traits in family studies is an important tool to identify genes that influence complex human diseases. The existing methods have severe limitations. In this article, we provide an efficient likelihood-based approach to investigating the associations between haplotypes and quantitative traits. Our approach acknowledges the ambiguities of haplotypes in the association analysis by integrating the construction of haplotypes and the estimation of haplotype effects into a single likelihood framework. In addition, our approach accommodates environmental factors, properly accounts for familiar correlations of trait values, and allows departures from Hardy-Weinberg equilibrium.

The proposed method appears to be more powerful than Haplotype FBAT, which is a conditional test. The haplotype FBAT, however, would be more robust to population stratification than the proposed method. To control for spurious association due to population stratification, one may partition the haplotype effect into between- and within-family components, as in [41, 42]. The between-family component accounts for all the spurious association and the within-family component provides a direct measure of the haplotype effect.

In this paper, we use the algorithm described in Zhang et al. [23] to identify the set of all possible haplotype configurations compatible with the observed genotype data. This set can be large for large pedigrees or when the number of SNPs under consideration is large. Although our theory applies to arbitrarily large pedigrees and large number of SNPs, the proposed method is computationally fast and numerically stable when both the pedigrees and the number of SNPs are small. To overcome the computational limitation, one can use the alternative haplotype reconstruction programs such as MERLIN, which only computes the most likely haplotype configuration. Future research is warranted to compare the statistical efficiency and computational efficiency of different haplotype reconstruction programs.

Some studies involve both families and unrelated individuals. Epstein et al. [43] proposed a likelihood-based approach to single-marker association analysis of binary traits using data from triads and unrelated subjects. For the haplotype-based association analysis of quantitative traits, our approach is applicable to arbitrary pedigrees and therefore can combine information from families and unrelated individuals in a single combined analysis. In fact, we can treat unrelated individuals as unrelated families but with just one individual in each family. This nice feature allows us to extract all available information and further improve the power of association tests.

Under model (2), the quantitative traits within a family follow a multivariate normal distribution. This model may not be appropriate for non-normal traits or traits with outliers. We are currently developing robust semiparametric variance-components models [44] for studying associations between haplotypes and non-normally distributed quantitative traits.

It is desirable to adjust for the effects of multiple testing when considering several haplotype configurations in the same study, especially in genomewide studies. The Bonferroni correction would be overly conservative and permutation would be computationally intensive. Huang et al. [45] proposed an efficient Monte-Carlo approach to adjusting for multiple testing for the haplotype analysis in case-control studies. It would be worthwhile to extend their approach to family studies.

In a haplotype association analysis, the set of all possible haplotype configurations can be large, even for a moderately large number of SNPs. Consequently, the number of parameters included in the model can be huge if we include all possible haplotype configurations in the phenotype model, and numerical computations may not be stable. We suggest specifying one or a few haplotypes of interest in the association test. We may consider a two-step procedure to identify a set of “risk" haplotypes. In the first step, for each possible haplotype configuration with the frequency above a certain threshold (e.g., 0.01), we fit the proposed model and estimate the haplotype effect. In the second step, we include those haplotypes with significant effects (e.g., with *p*-values <0.05) in the phenotype model. This procedure is similar to some methods in the variable selection literature. It would be interesting to investigate the properties of such a procedure in the future.

In the presence of missing genotype data, one can carry out the single-SNP analysis by using the imputation method, in particular, the multiple imputation procedure [46]. In contrast, the proposed method is based on the full information maximum likelihood, which tends to have more efficient parameter estimators than the multiple imputation method when the model assumptions are satisfied. Additionally, multiple imputation method requires repeated runs of the model, and special care is needed to estimate the standard errors of the parameter estimators. On the other hand, the multiple imputation method is more flexible than the full information maximum likelihood approach.

One limitation of the proposed method is that it is developed for common variant analysis. With the availability of high-throughput sequencing data, it would be interesting to develop rare variance analysis methods for family studies with missing data. This is a topic for future research.

## Methods

Suppose that the study contains *n* families or general pedigrees, with *n*_*i*_ individuals in the *i*th pedigree. Let *Y*_*ij*_ be the quantitative trait of interest and **x**_*ij*_ be a set of environmental variables for the *j*th member of the *i*th pedigree. Assume that each individual is genotyped at *M* tightly linked diallelic SNPs. At each SNP locus, the two possible alleles are denoted by 0 and 1. The total number of possible haplotypes is *K*=2^*M*^. For example, the possible haplotypes for three SNPs are 000, 001, 010, 011, 100, 101, 110, and 111. For *k*=1,⋯,*K*, let *h*_*k*_ denote the *k*th possible haplotype. The distribution of the diplotype (i.e., the pair of haplotypes on the two homologous chromosomes) is often assumed to satisfy Hardy-Weinberg equilibrium such that

$$\pi_{kl}=\pi_{k} \pi_{l}, \text{} k,l=1,\cdots,K, $$

where *π*_*kl*_ is the probability that the diplotype *H* consists of *h*_*k*_ and *h*_*l*_, and *π*_*k*_ is the population frequency of haplotype *h*_*k*_. We consider the following extension of Hardy-Weinberg equilibrium:

$$\pi_{kl}= \left\{\begin{array}{ll} \pi_{k}^{2}/(1-\rho+\rho\sum_{j=1}^{K}\pi_{j}^{2}), & k=l, \\ (1-\rho)\pi_{k}\pi_{l}/(1-\rho+\rho\sum_{j=1}^{K}\pi_{j}^{2}), &k\neq l, \\ \end{array}\right. $$

where $0\leq \pi _{k}\leq 1, \sum _{k=1}^{K}\pi _{k}=1$, and *ρ* is the inbreeding coefficient [47, p. 93]. Excessive homozygosity and excessive heterozygosity arise under *ρ*>0 and *ρ*<0, respectively. The special case of *ρ*=0 corresponds to Hardy-Weinberg equilibrium.

For *i*=1,…,*n* and *j*=1,…,*n*_*i*_, let *H*_*ij*_≡(*H*_*ij*1_,*H*_*ij*2_) denote the diplotype of the *j*th member of the *i*th family, and *G*_*ij*_ denote the corresponding multi-locus genotype. Note that *G*_*ij*_ codes the number of the “1” allele at each locus such that *G*_*ij*_=*H*_*ij*1_+*H*_*ij*2_. We cannot determine *H*_*ij*_ from *G*_*ij*_ with certainty if the individual is heterozygous at more than one SNP site or if any SNP genotype is missing.

In association studies, we are interested in estimating the effects of *H*_*ij*_ and **x**_*ij*_ and possibly their interactions on *Y*_*ij*_. However, we observe *G*_*ij*_ instead of *H*_*ij*_. We denote the probability distribution of *H*_*ij*_ by *P*(*H*_*ij*_;***γ***), where ***γ*** consists of *π*_*k*_(*k*=1,⋯,*K*) and *ρ*. By using a haplotype reconstruction program, such as HAPLORE [23], we can identify the set of all possible haplotype configurations, denoted by *S*(**G**_*i*_), which are compatible with (possibly missing) genotype $\phantom {\dot {i}\!}\mathbf {G}_{i}\equiv (G_{i1},\cdots, G_{in_{i}})$.

We specify the following linear regression model with random effects

2 $$ Y_{ij} = \alpha + {\boldsymbol{\beta}}^{\mathrm{T}}\mathbf{Z}(H_{ij1},H_{ij2},\mathbf{x}_{ij}) + g_{ij} + e_{ij},  $$

where *α* is the intercept, **Z**(*H*_*ij*1_,*H*_*ij*2_,**x**_*ij*_) is a vector function of (*H*_*ij*1_,*H*_*ij*2_) and **x**_*ij*_,***β*** is the corresponding set of regression parameters, *g*_*ij*_ is a random effect due to genes at unlinked loci, and *e*_*ij*_ is an individual-specific residual environmental effect. Note that *g*_*ij*_ is used to capture the correlations of the quantitative trait values within the family. The random variables *g*_*ij*_ and *e*_*ij*_ are assumed to be independent zero-mean normal with variances $\sigma _{g}^{2}$ and $\sigma _{e}^{2}$, respectively. The phenotypic covariance matrix of $\phantom {\dot {i}\!}\mathbf {Y}_{i} \equiv (Y_{i1}, \cdots, Y_{in_{i}})^{\mathrm {T}}$ can be expressed as

$$\mathbf{V}_{i} = 2\sigma_{g}^{2} {\boldsymbol{\Sigma}}_{gi} + \sigma_{e}^{2} \mathbf{I}_{i}, $$

where ***Σ***_*gi*_ is the matrix of kinship coefficients, and **I**_*i*_ is an identity matrix.

We define **Z**(*H*_*ij*1_,*H*_*ij*2_,**x**_*ij*_) according to the genetic mode of inheritance. For example, the choice of

$$\mathbf{Z}(H_{ij1}, H_{ij2},\mathbf{x}_{ij}) = \left[ \begin{array}{c} I(H_{ij1}=h^{*})+I(H_{ij2}=h^{*})\\ \mathbf{x}_{ij}\\ \{I(H_{ij1}=h^{*}) +I(H_{ij2}=h^{*})\}\mathbf{x}_{ij} \end{array} \right]$$

corresponds to an additive model for the haplotype effect, environmental effects and haplotype-environment interactions, where *h*^∗^ is the target haplotype of interest, and *I*(·) is the indicator function. If we are interested in the recessive or dominant effect of *h*^∗^, then we set the genotype score in **Z**(*H*_*ij*1_,*H*_*ij*2_,**x**_*ij*_) to *I*(*H*_*ij*1_=*H*_*ij*2_=*h*^∗^) or *I*(*H*_*ij*1_=*h*^∗^ or *H*_*ij*2_=*h*^∗^), respectively. We may include additional terms in **Z** so as to assess the effects of several haplotype configurations and to test for multi-haplotype effects.

Write ${\boldsymbol {\theta }}=(\alpha, {\boldsymbol {\beta }}^{\mathrm {T}}, \sigma _{g}^{2}, \sigma _{e}^{2})^{\mathrm {T}}$. Let *P*(**Y**_*i*_|**x**_*i*_,**H**_*i*_;***θ***) denote the multivariate normal density function of **Y**_*i*_ conditional on **x**_*i*_ and **H**_*i*_, where $\phantom {\dot {i}\!}\mathbf {x}_{i}=(\mathbf {x}_{i1},\cdots, \mathbf {x}_{in_{i}})$, and $\phantom {\dot {i}\!}\mathbf {H}_{i}=(H_{i1},\cdots,H_{in_{i}})$. Let *P*(**H**_*i*_;***γ***) denote the probability distribution of **H**_*i*_. Note that *P*(**H**_*i*_;***γ***) is proportional to

$$\prod_{j\in \{\text{founders}\}} P(H_{ij};{\boldsymbol{\gamma}}).$$

The complete-data likelihood function for parameters ***θ*** and ***γ*** given (**Y**_*i*_,**x**_*i*_,**H**_*i*_,**G**_*i*_)(*i*=1,⋯,*n*) is proportional to

$$\prod_{i=1}^{n} P(\mathbf{Y}_{i} |\mathbf{x}_{i}, \mathbf{H}_{i};{\boldsymbol{\theta}}) P(\mathbf{H}_{i};{\boldsymbol{\gamma}}), $$

and the likelihood function based on the observed data (**Y**_*i*_,**x**_*i*_,**G**_*i*_)(*i*=1,⋯,*n*) is proportional to

3 $$ L({\boldsymbol{\theta}},{\boldsymbol{\gamma}}) = \prod_{i=1}^{n} \sum_{\mathbf{H}_{i} \in S(\mathbf{G}_{i})} P(\mathbf{Y}_{i} |\mathbf{x}_{i}, \mathbf{H}_{i};{\boldsymbol{\theta}}) P(\mathbf{H}_{i};{\boldsymbol{\gamma}}).  $$

It is worth noting, although we use the algorithm in HAPLORE to identify the set *S*(**G**_*i*_), we maximize the likelihood in (3) to obtain the estimators of all unknown parameters simultaneously, including haplotype frequencies, regression coefficients, and variance parameters.

Treating the *H*_*ij*_’s as missing data, we maximize (3) through the EM algorithm. In the (*t*+1)th E-step, we calculate the conditional expectation of the logarithm of (3) given the observed data and current parameter estimates $\widehat {{\boldsymbol {\theta }}}^{(t)}$ and $\widehat {{\boldsymbol {\gamma }}}^{(t)}$ as follows:

4 $$ \sum_{i=1}^{n} \sum_{\mathbf{H}_{i}\in S(\mathbf{G}_{i})} \omega_{\mathbf{H}_{i}}^{(t)}\left\{ \log P(\mathbf{Y}_{i}|\mathbf{x}_{i}, \mathbf{H}_{i};{\boldsymbol{\theta}}) + \log P(\mathbf{H}_{i};{\boldsymbol{\gamma}})\right\},  $$

where

$$\omega_{\mathbf{H}_{i}}^{(t)} = \frac{P(\mathbf{Y}_{i}|\mathbf{x}_{i},\mathbf{H}_{i};\widehat{{\boldsymbol{\theta}}}^{(t)})P(\mathbf{H}_{i};\widehat{{\boldsymbol{\gamma}}} ^{(t)})}{\sum_{\mathbf{H}_{i}^{*}\in S(\mathbf{G}_{i})} P(\mathbf{Y}_{i}|\mathbf{x}_{i}, \mathbf{H}_{i}^{*};\widehat{{\boldsymbol{\theta}}}^{(t)})P(\mathbf{H}_{i}^{*};\widehat{{\boldsymbol{\gamma}}} ^{(t)})}, $$

which is the conditional probability that the haplotype configuration for the *i*th family is **H**_*i*_ given the observed data and current parameter estimates. In the (*t*+1)th M-step, we maximize (4) to update parameter estimates. We iterate the E-step and the M-step until convergence. One can also maximize the observed-data likelihood function (3) directly by using an optimization algorithm [48]. The resultant maximum likelihood estimator (MLE) is denoted by $(\widehat {{\boldsymbol {\theta }}},\widehat {{\boldsymbol {\gamma }}})$. By extending the arguments of Lin and Zeng [21] and Diao and Lin [44], we can show that $(\widehat {{\boldsymbol {\theta }}},\widehat {{\boldsymbol {\gamma }}})$ is consistent, asymptotically normal and asymptotically efficient. In addition, the covariance matrix of $(\widehat {{\boldsymbol {\theta }}},\widehat {{\boldsymbol {\gamma }}})$ can be estimated by the inverse of the observed information matrix. To test the haplotype effects or haplotype-environment interactions, we calculate the likelihood ratio test statistic

$$LR = -2[\log L(\widetilde{{\boldsymbol{\theta}}},\widetilde{{\boldsymbol{\gamma}}}) - \log L(\widehat{{\boldsymbol{\theta}}},\widehat{{\boldsymbol{\gamma}}})], $$

where $(\widetilde {{\boldsymbol {\theta }}},\widetilde {{\boldsymbol {\gamma }}})$ is the restricted MLE under the null hypothesis. The null distribution of *LR* is asymptotically *χ*^2^ with the degrees of freedom equal to the number of the regression parameters in the null hypothesis. (Note that we are testing the regression effects, not the variance components.) The Wald statistic can also be used to perform hypothesis testing and construct confidence intervals.

Analysis of single SNPs with missing genotypes can be treated as a special case of the proposed haplotype analysis. If we are interested in the additive effect of a particular SNP, then we set the genotype score in *Z*(*H*_*ij*1_,*H*_*ij*2_,**x**_*ij*_) in model (2) to be the value of (*H*_*ij*1_+*H*_*ij*2_) at that SNP position; recessive and dominant effects can be similarly modeled. We can also define *Z*(*H*_*ij*1_,*H*_*ij*2_,**x**_*ij*_) to formulate the joint effects of all *M* SNPs or any subset of them.

When one of the *M* SNPs is untyped, there is no information in the study data to estimate the joint distribution of the *M* SNPs. We can infer the joint distribution from an external reference database, such as the HapMap. Naturally, the family study and the reference panel are assumed to come from a common population. Let *L*_*R*_(***γ***) denote the likelihood for ***γ*** based on the reference database. Then the likelihood for (***θ***,***γ***) that combines the study data and reference database is

$$L_{C}({\boldsymbol{\theta}},{\boldsymbol{\gamma}})=L({\boldsymbol{\theta}},{\boldsymbol{\gamma}}) L_{R}({\boldsymbol{\gamma}}). $$

The EM algorithm described earlier can be used to maximize the likelihood *L*_*C*_(***θ***,***γ***). The resultant MLE of (***θ***,***γ***) preserves the desired asymptotic properties.

We have developed a stand-alone computer program that implements the new methods. The program is reasonably efficient in terms of computation. It takes about 0.8 seconds to analyze one data set in the simulation studies presented in the next section on an iMac with a 3 GHz Intel Core i5 processor. This program is freely available on the website: https://sites.google.com/view/guoqingdiao-homepage.

## Data Availability

Data used in this article comes from the Childhood Asthma Management Program Ancillary Genetic Study.
